# Light manipulation of mosquito behaviour: acute and sustained photic suppression of biting activity in the *Anopheles gambiae* malaria mosquito

**DOI:** 10.1186/s13071-017-2196-3

**Published:** 2017-06-16

**Authors:** Aaron D. Sheppard, Samuel S. C. Rund, Gary F. George, Erin Clark, Dominic J. Acri, Giles E. Duffield

**Affiliations:** Department of Biological Sciences and Eck Institute for Global Health, Galvin Life Science Center, University of Notre Dame, Notre Dame, IN 46556 USA

**Keywords:** *Anopheles gambiae*, Behaviour, Biting, Blood-feeding, Circadian rhythm, Flight activity, Light, Locomotor activity, Malaria, Mosquito, Photic, Physiology

## Abstract

**Background:**

Host-seeking behaviours in anopheline mosquitoes are time-of-day specific, with a greater propensity for nocturnal biting. We investigated how a short exposure to light presented during the night or late day can inhibit biting activity and modulate flight activity behaviour.

**Results:**

*Anopheles gambiae* (*s.s*.), maintained on a 12:12 LD cycle, were exposed transiently to white light for 10-min at the onset of night and the proportion taking a blood meal in a human biting assay was recorded every 2 h over an 8-h duration. The pulse significantly reduced biting propensity in mosquitoes 2 h following administration, in some trials for 4 h, and with no differences detected after 6 h. Conversely, biting levels were significantly elevated when mosquitoes were exposed to a dark treatment during the late day, suggesting that light suppresses biting behaviour even during the late daytime. These data reveal a potent effect of a discrete light pulse on biting behaviour that is both immediate and sustained. We expanded this approach to develop a method to reduce biting propensity throughout the night by exposing mosquitoes to a series of 6- or 10-min pulses presented every 2 h. We reveal both an immediate suppressive effect of light during the exposure period and 2 h after the pulse. This response was found to be effective during most times of the night: however, differential responses that were time-of-day specific suggest an underlying circadian property of the mosquito physiology that results in an altered treatment efficacy. Finally, we examined the immediate and sustained effects of light on mosquito flight activity behaviour following exposure to a 30-min pulse, and observed activity suppression during early night, and elevated activity during the late night.

**Conclusions:**

As mosquitoes and malaria parasites are becoming increasingly resistant to insecticide and drug treatment respectively, there is a necessity for the development of innovative control strategies beyond insecticide-treated nets (ITNs) and residual spraying. These data reveal the potent inhibitory effects of light exposure and the utility of multiple photic pulses presented at intervals during the night/late daytime, may prove to be an effective tool that complements established control methods.

## Background

Malaria infects over 250 million people and claims the lives of approximately 438,000 individuals annually. Approximately 90% of these deaths occur in Africa, predominantly in children aged 5 years and under [1]. *Anopheles gambiae* is the major vector of malaria in sub-Saharan Africa. Current control of malaria transmission is through control of the insect vector and efforts to reduce biting events that can result in disease transmission. As the female anopheline mosquito focuses its behavioural activity including blood-feeding to the night phase of the light:dark (LD) cycle, the mosquito bed net has become a key barrier method in malaria transmission control. However, it is fast becoming apparent that the efficacy of established methods of control, namely insecticide-treated bed nets (ITNs) and indoor residual spraying (IRS) are compromised by both resistance to insecticides [2, 3] and by behavioural adjustments made presumably *via* changes in the genetic composition of the *Anopheles* vector mosquito populations [4–11]. It has been observed in various anophelines that the specific timing of biting activity can change under selective pressure, thereby resulting in a temporal shift. This can result in more biting events occurring either earlier in the night, dawn or during the early daytime (i.e. becoming daytime biters), and thereby make the efficacy of the bed net significantly reduced. It is for this reason that efforts are being made by the vector research community to develop new strategies to complement existing control methods, including control of outdoor residual mosquito populations. In line with this approach, we explored the efficacy of timed exposure to light as a potential method to reduce biting behaviour in *An. gambiae* mosquitoes.

There is a limited but established body of work exploring how light impacts aspects of mosquito behaviour, and in particular that of the *An. gambiae* circadian timing system (reviewed in [12, 13]). Photic cues can shape the 24-h profiles of gene expression [14, 15] and flight activity of *An. gambiae* mosquitoes [16–18]. However, there is little work to date that has explored how light can modulate mosquito biting behaviour. Das & Dimopoulos [19] examined the effect of discrete treatments of white light presented during the late night phase of the diel cycle on biting activity using a membrane feeding system and testing an outbred strain of *An. gambiae* [19]. Under these specific experimental conditions, it was revealed that as little as 2 min of 800–1000 lux light has a potent inhibitory effect on blood-feeding activity when presented at this specific time of the night and that inhibition can be measured for up to 2 h after the pulse. In the current investigation, we explored the efficacy of timed exposure to white light to modulate biting activity using a human-subject biting assay. The study focused on examining a broad range of times across the diel cycle, including the early night and during the late day-time, times of the LD cycle when humans may be unprotected by bed nets. To complement the biting behaviour assays, we also explored how light might alter flight activity, as it is a component of host-seeking behaviour. Previous work in our laboratory and by Das & Dimopoulos [19] revealed a 24-h diel rhythm in biting activity that also persists under constant conditions of darkness, thereby demonstrating that the rhythm is in part shaped by the mosquito endogenous circadian clock [19, 20]. Differences between the biting profiles examined under LD cycle and constant dark conditions also provided evidence for light exposure during the light phase of the natural diel cycle to affect blood-feeding propensity [20]. It was these data on diel and circadian biting rhythms that provided the impetus for the current investigation, and we hypothesised that a discrete treatment of light would reproducibly suppress *An. gambiae* blood-feeding behaviour and modulate flight activity.

In this study, we have developed a technique to reduce the incidence of biting and to disrupt the normal profile of nocturnal flight activity of the anopheline mosquito. Using exposure to white light presented at timed intervals during the late daytime, dusk, dawn, and during the night, we have demonstrated dramatic reductions in mosquito biting of humans as well as increased or decreased levels of mosquito flight activity, dependent upon the specific timing of light delivery. To reduce mosquito host-seeking and biting events, and thereby reduce malaria transmission, we propose that the photic exposure method could be used to augment current insect control techniques or be implemented as a stand-alone approach.

## Methods

### Mosquito rearing

**Fig. 1:**
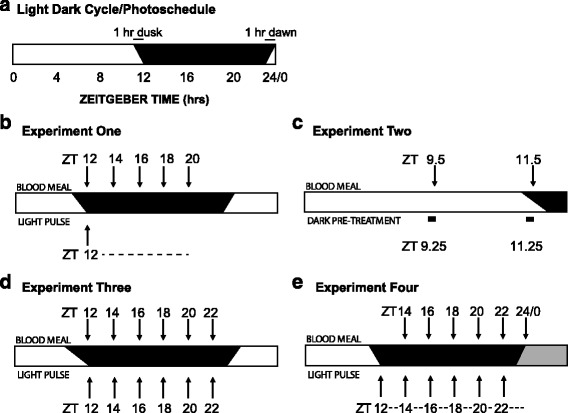
Schematic representation of the biting behavioural assays. **a** Standard photoschedule that the *An. gambiae* (*s.s*.) mosquitoes were reared on and experimented under. Zeitgeber time (ZT) 12 is the time of lights off, and ZT24/0 occurs at the end of the 1 h dawn transition. **b** Method for testing the immediate (acute) and sustained (chronic) effects of a single light pulse presented during the onset of the night on biting activity. The dotted line represents a single, one-time light pulse for all experimental mosquitoes. **c** Method for testing the effects of dark acclimation on blood-feeding behaviour during the late daytime and the dusk transition. **d** Method for assaying the immediate acute inhibition of biting behaviour by a single light pulse tested at different times throughout the night. **e** Method for testing the sustained (chronic) inhibition of biting behaviour 2 h following light treatment and with multiple light pulses delivered throughout the night. Each batch of mosquitoes was assayed for blood-feeding in the dark immediately prior to the next time series batch being exposed to light. The dotted line represents all experimental mosquitoes were light-pulsed repeatedly. White-black bar represents the environmental LD cycle. A grey section (**e**) represents subjective day when the LD cycle ends in the experiment, and there is a transition to constant darkness. Arrows directly above the light-dark bar pointing down indicate the time of blood meal (Investigator’s arm) being offered (**b**, **c**, **d**, **e**); arrows directly below the light-dark bar pointing up to indicate the time specific presentation of white light (**b**, **d**, **e**). A black box below the light-dark bar indicates the administration of a pretreatment of darkness during the light phase of the LD cycle (**c**)

Female *An. gambiae* (*s.s*.) (S form) Pimperena strain mosquitoes (MRA-861, BEI Resources, Manassas, VA, USA) were used for the biting light-inhibition and locomotion/flight activity assays. Note that this strain has been utilized by us [14, 16, 20, 21] and others [22–24] in the laboratory for several years; this was one of the colonies used for the S- *versus* M- molecular form sequencing project [25] (now reclassified as *An. gambiae* (*s.s*.) and *An. coluzzii*, respectively), and is available through the MR4 NIAID/NIH BEI Resource Repository. Mosquitoes were maintained at 27 ± 1 °C and 85 ± 1% humidity on a 12:12 LD cycle (11 h ~ 250 lux full light, 11 h darkness, and 1 h dawn and dusk transitions) (Fig. 1a). Reported times when mosquitoes are examined under LD cycle conditions are recorded as Zeitgeber time (ZT) with ZT0 being defined as the end of the 1 h dawn transition and the beginning of the full light cycle, and ZT12 as the onset of the dark phase. In experiments in which mosquitoes are transitioned from a LD cycle into constant darkness at the end of the normal night/dark phase, time is reported thereafter in Circadian Time (CT), with CT0 being the start of the subjective day and CT10 being the late subjective day. A 20% high fructose corn syrup solution was available *ab libitum* throughout all experiments; the taking of blood-meals occurred only in a light, temperature and humidity controlled bay (a walk-in incubator connected to an anteroom) and in the dark phase of the LD cycle unless otherwise stated. The assessment of blood meals occurred in the anteroom. Experiments were conducted on 4–10 day old presumed mated female mosquitoes (i.e. host-seeking). Additionally, unless otherwise stated, all mosquitoes used for biting assays, once transferred into experimental containers, were placed in plastic light tight boxes (1 m [L] × 0.6 m [W] × 0.5 m [H]) with their individual lighting systems. These boxes were used to mimic the LD cycle of the rearing bay as well as deliver the light pulse protocols as described below.

### Light-inhibition biting assays

#### Experiment 1: Single light pulse biting inhibition assay during early night

To test for light-induced acute (immediate) and chronic (sustained) biting inhibition, ≥ 25 mosquitoes were placed into each of 10 small white translucent plastic housing containers (8–10 cm diameter × 10 cm tall with a 64 cm^2^ gauze mesh screen covering the top of the container) ≥ 24 h before experimentation. Five containers received a 10 min light pulse (300 lux [intensity equivalent to comfortable human reading conditions] at ZT12) provided by a LED light bar (Utilitech GU0018P-LED-1, Good Earth Lighting, Inc., Wheeling, IL, USA); the other 5 containers entered the dark phase of the LD cycle and received no light exposure (controls). Immediately following the light pulse treatment (i.e. ZT12:10), one light-pulsed and one control container were simultaneously offered a blood meal for 6 min in the dark (an Investigator’s arm was placed across the mesh at the top of the container) [20]. Blood meals were offered to subsequent containers at ZT14, 16, 18, and 20 (Fig. 1b). It is important to note that for this approach each experimental container of mosquitoes only received a single light treatment. At the end of the blood-feeding period, the mosquitoes were then anaesthetized with CO_2_ and visually assayed for the presence of blood in the abdomen (i.e. the abdomen visually appeared to be coloured red when viewed by eye, with backlight illumination, indicating a partially or fully fed mosquito). Four replicate experiments were conducted.

#### Experiment 2: Late diel day, dark-acclimated biting assay

To investigate endogenous biting behaviour during the late daytime and the effect of light during the natural LD cycle, a pair of small housing containers each containing ≥ 25 mosquitoes were exposed to either a single 15 min dark pre-treatment (experimental mosquitoes), or remained on the normal LD cycle (control mosquitoes) prior to the pair simultaneously being offered a blood meal for 6 min in the dark (Investigator’s arm). This assay was performed with one pair of containers of mosquitoes blood-feeding at 1.5 h prior to dusk/2.5 h prior to lights off (ZT9.5), and another pair of containers during the dusk transition/30 min before lights off (ZT11.5). Following exposure, the mosquitoes were visually assayed for the presence of blood in the abdomen (Fig. 1c). Five replicate experiments were conducted.

#### Experiment 3: Single-pulse acute biting inhibition assay

To examine the acute (immediate) inhibition effect of light on biting behaviour at different times during the night, and to determine whether this may change as a function of diel/circadian time, 12 large white translucent containers (17–19 cm diameter × 17 cm tall with a 100 cm^2^ mesh screen), containing ≥ 30 mosquitoes were prepared prior to experimentation. At each time point (ZT12, 14, 16, 18, 20 and 22) one experimental container received a 6 min white light pulse (150 lux; illuminated from the base of the container) while simultaneously being offered a blood meal from the top of the container. The control container of mosquitoes was not exposed to light and was offered a blood meal for 6 min while in the dark. As experimental (exposed to light) and control (in the dark) containers of mosquitoes could not be fed simultaneously, experimental and control containers were fed in series one immediately after the other, and alternating the order of feeding at each time point. It is important to note that each experimental container of mosquitoes only received a single light treatment. At the end of the 6 min exposure to the Investigator’s arm the number of mosquitoes having taken a blood meal was recorded as described above (Fig. 1d). Three replicate experiments were conducted.

#### Experiment 4: Multi-pulse chronic biting inhibition assay

To examine the chronic (sustained) light suppression of biting *via* the administration of multiple light pulses, 12 small containers of ≥ 25 mosquitoes were prepared. Six containers (experimental groups) received a white light pulse (300 lux) for 10 min at ZT12, while the remaining six housing containers began the dark cycle and received no light exposure (control). At ZT14, one experimental and one control container were removed and simultaneously offered a 6 min blood meal in the dark, and the number of mosquitoes that took a blood meal was recorded as previously described. At ZT14, the 5 remaining experimental containers received another 10 min light pulse. This procedure of pulsing and blood-feeding was repeated at ZT16, 18, 20, and 22 (with the last blood meal being offered at CT24/CT0 and where the dawn transition and lights on did not take place for either experimental or control mosquitoes) (Fig. 1e). It is important to note that in this approach each experimental container of mosquitoes, except for the ZT14 group, received multiple light pulses during the night, and for each set of mosquitoes, a single opportunity to blood feed, i.e. the ZT16 group received 2 pulses; ZT18, 3 pulses; ZT20, 4 pulses; and the ZT22 group received a total of 5 pulses. Four replicate experiments were conducted.

#### Experiment 5: Light manipulation of locomotion assay

Mosquitoes were individually loaded into 32, 15 cm long × 2.5 cm diameter glass test tubes. The end of each test tube was plugged using cotton wool wrapped around a 2 ml microcentrifuge tube containing 20% high fructose corn syrup, providing *ad libitum* access to sugar water for the duration of the experiment. Test tubes were then placed in Locomotion Activity Monitors 25 (LAMs; TriKinetics, Waltham, MA, USA) and locomotor/flight activity recorded. A photon-detector at the centre of the tube was activated any time the mosquito moved across an infrared beam of light, and the number of crossings in a 1 min period was recorded. LAMs were transferred to plastic light tight boxes as described above (see [16] for additional experimental setup information). An identical 12:12 LD cycle with 1 h dawn and dusk transitions within the box was matched to the rearing bay of the insectary for 4 days before transitioning to constant dark (DD) conditions (Fig. 1a). At the end of the forth full 24 h day and during the first 12 h in DD (i.e the transition to the first circadian cycle), mosquitoes were exposed to a 30 min white light pulse (300–870 lux, measured at the bottom to top of LAM unit) delivered automatically from within the box at either ZT 12, 14, 15, 16, 18, 20, or 22, or CT 24/0, 2, 4, 6, or 8. Note that the activity recorded during the day that the mosquitoes were first introduced to the LAM unit (Day 1) was excluded from analysis to allow for stabilisation of behaviour, and in instances, it contained an incomplete 24 h. Recording of activity under DD conditions continued for 10 days after the light pulse was delivered. The locomotion data were analysed to determine whether light pulses perturbed flight behaviour in mosquitoes acutely and chronically. The average number of beam crossings (counts) during the 30 min light pulse on the pulse day was determined, and compared to the counts during the matching Zeitgeber times of the prior two control days. For a sustained/chronic effect of light analysis, activity was also examined for the 3 days (pulse day and prior 2 days) at different intervals over an 8 h period that followed immediately after the light pulse ended. Sixty minutes of activity was analysed specifically during the first, second, third, fourth and eighth hour following cessation of the 30 min light pulse. The average number of beam breaks during the 1 h period at the different intervals was determined and the equivalent Zeitgeber times on the 3 days compared. The sustained/chronic effect analysis was performed on data from pulses delivered at ZT12, 16, 22 and CT24/0, times where acute effects of the pulse were observed.

### Statistical analysis

Statistical analysis was performed using Sigma-Plot 12 (Systat Software, Chicago, IL, USA) and/or Microsoft Excel (Microsoft, Redmond, WA, USA). Biting percentages and locomotion/flight activity were compared for statistical differences by one-way ANOVA, two-way ANOVA, repeated-measures ANOVA, and followed by Tukey *post*-*hoc* tests where appropriate. Statistical significance was at the level of *P* < 0.05. In the time-specific flight activity experiments, changes in activity were only reported as significant if the treatment day was found different in the *post*-*hoc* analysis to both of the preceding control days.

## Results

### Experiment 1: Biting behaviour can be suppressed by a pulse of light delivered during the early night

**Fig. 2:**
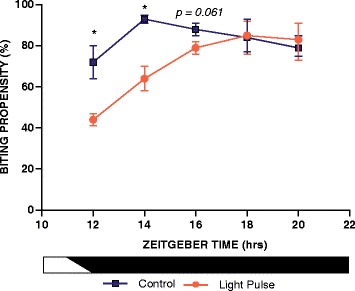
Experiment 1: Single light pulse treatment revealed immediate (acute) and sustained (chronic) suppression of biting. Representative biting percentages of mosquitoes either after receiving a single 10 min, ~300 lux white light pulse at ZT12 or transitioning normally into the constant dark (control treatment). Light and control treated mosquitoes were simultaneously offered a blood meal for 6 min at ZT12.2 and then again at ZT14, 16, 18, and 20. The percentage of mosquitoes per container that blood-fed was recorded immediately after the blood meal was offered by observing blood in the abdomen. Two-way ANOVA (effect of treatment, *F*_(1,35)_ = 13.8, *P* < 0.001; effect of ZT, *F*_(4,35)_ = 11.6, *P* < 0.001; interaction, *F*_(4,35)_ = 3.7, *P* < 0.05) followed by Tukey *post*-*hoc* tests (*P < 0.05). Values shown are mean ± SEM

To determine whether light presented to mosquitoes during the early night would suppress biting behaviour, populations of female *An. gambiae* (*s.s*.) mosquitoes were tested with a human biting assay in the dark. Mosquitoes were treated with a light pulse at the beginning of the dark phase/end of the dusk transition (ZT12), and biting propensity was recorded immediately after presentation of the light and at 2-h intervals by examining mosquitoes for the presence of blood (Fig. 2). Light-treated *An. gambiae* exhibited a reduced biting propensity compared to time-matched controls, specifically showing reduced biting by 42% of control levels immediately following the light pulse (ZT12:10) and by 32% 2 h post light pulse (ZT14). Of note, biting propensity 4 h following treatment appeared to be reduced but did not reach significance level (ZT16; *P* = 0.061); however, the significance value is low enough to warrant mention.

### Experiment 2: Increased biting propensity from exposure to darkness (dark pre-treatment) during the late daytime

**Fig. 3:**
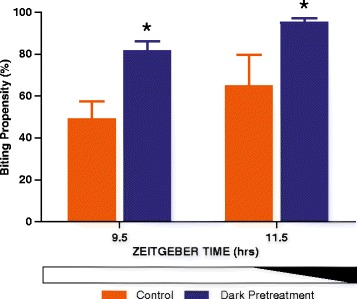
Experiment 2: Mosquitoes exposed to darkness during the late daytime exhibit increased biting activity. Mosquito biting behaviour is increased after receiving a 15 min dark pretreatment when tested at two different times of the late day including during the dusk transition. Blood meals were offered at ZT9.5 and ZT11.5 in the dark following either a dark pretreatment or normal exposure to light of the LD cycle and dusk transition (controls). Dusk progression is indicated by the horizontal white/black bar occurring at ZT11-12. Two-way ANOVA (effect of treatment, *F*_(1,15)_ = 12.0, *P* = 0.003; effect of ZT, *F*_(1,15)_ = 2.6, *P* = 0.125; interaction, *F*_(1,15)_ = 0.02, *P* = 0.907), followed by *post*-*hoc* tests (*P < 0.05). Values shown are mean ± SEM

Our previous diel and circadian analysis of biting propensity using the same 6 min exposure to a human arm blood-meal/biting assay, revealed not only a distinct rhythm in biting behaviour, peaking during the dark phase of the LD cycle or during subjective night, but also alluded to the possibility that light itself presented during the circadian day/light phase of the LD cycle could suppress biting [20]. To further evaluate in a quantifiable manner this light exposure property during the late daytime, mosquitoes were dark adapted by a 15 min pretreatment of darkness starting at ZT9.25 or ZT11.25 and assessed for biting propensities at ZT9.5 and ZT11.5, respectively. This treatment during the late daytime or during dusk resulted in a 58% elevation in biting propensity for dark-adapted mosquitoes compared to those maintained on the regular LD cycle. This effect was observed regardless of the time of testing, and the magnitude of increased biting at each time point was similar (Fig. 3). These data, therefore, reveal that light suppression of biting can occur not only during the night phase but also during the day phase of the diel cycle.

### Experiment 3: Light can immediately suppress biting activity when presented at almost all phases of the night

**Fig. 4:**
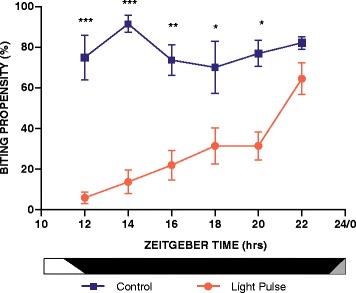
Experiment 3: Immediate (acute) inhibition of biting behaviour by a single light pulse tested at different times of the diel night. Inhibition of blood-feeding in mosquitos that were blood-fed in the light *versus* fed in the dark. The inhibitory effect of light on blood-feeding declined as the dark period progressed. Blood meals were offered during the biological night at ZT12, 14, 16, 18, 20 and 22 under either light or dark conditions. Experimental mosquitoes were subject to a single light pulse while simultaneously being blood-fed. Two-way ANOVA (effect of treatment, *F*_(1,32)_ = 50.6, *P* < 0.001; effect of ZT, *F*_(5,32)_ = 2.2, *P* = 0.097; interaction, *F*_(5,32)_ = 1.8, *P* = 0.161), followed by *post*-*hoc* tests (*P <0.05, **P <0.01 and ***P <0.001). Values are mean ± SEM

Due to the efficacy of the single light treatment in reducing biting behaviour in an immediate manner (Fig. 2), we evaluated whether such a discrete light treatment would have similar efficacy when presented at different times of the night. We, therefore, examined the acute biting inhibition effect at 2-h intervals throughout the night, and where mosquitoes were exposed to light at the actual time of exposure to the human arm. Light-treated *An. gambiae* mosquitoes showed a significant and dramatic decrease in biting propensity (55–94%) compared to controls, which was consistently observed throughout the night (Fig. 4). Tukey *post*-*hoc* analysis revealed differences at all times tested except ZT22. Interestingly, while biting of control non-pulsed mosquitoes showed high biting levels throughout the night, and with the highest levels at ZT14, the degree of inhibition became progressively less as the night progressed and in a stepwise progression (Fig. 4). This reduction of biting propensity is indeed reflected by the diminishing significance levels in the time-specific *post*-*hoc* tests, i.e. at ZT12 (*P* < 0.001), ZT14 (*P* < 0.001), ZT16 (*P* = 0.005) ZT18 (*P* = 0.025) and ZT20 (*P* = 0.017), but not ZT22 (*P* = 0.498). While no significant difference was observed between treated and non-pulsed mosquitoes at ZT22 (1 h before the start of dusk), a 23% reduction was observed. These data suggest a circadian regulation of immediate light suppression of biting behaviour that achieves the greatest efficacy during the early night and the least efficacy during late night. Furthermore, this immediate suppression of biting during the exposure to light appears to be a stronger suppressor than when assessed immediately after the presentation of light (Figs. 2 and 4): at ZT12, biting propensity is suppressed dramatically by as much as 94% of the mosquito population when assayed during the light treatment, whereas 42% of the population show biting suppression when tested in the dark immediately after light treatment. Nevertheless, the after-effect of photic stimulation measured at ZT12 is still a large change in behaviour.

### Experiment 4: Exposure to repeated pulses of light result in sustained biting inhibition during the night

**Fig. 5:**
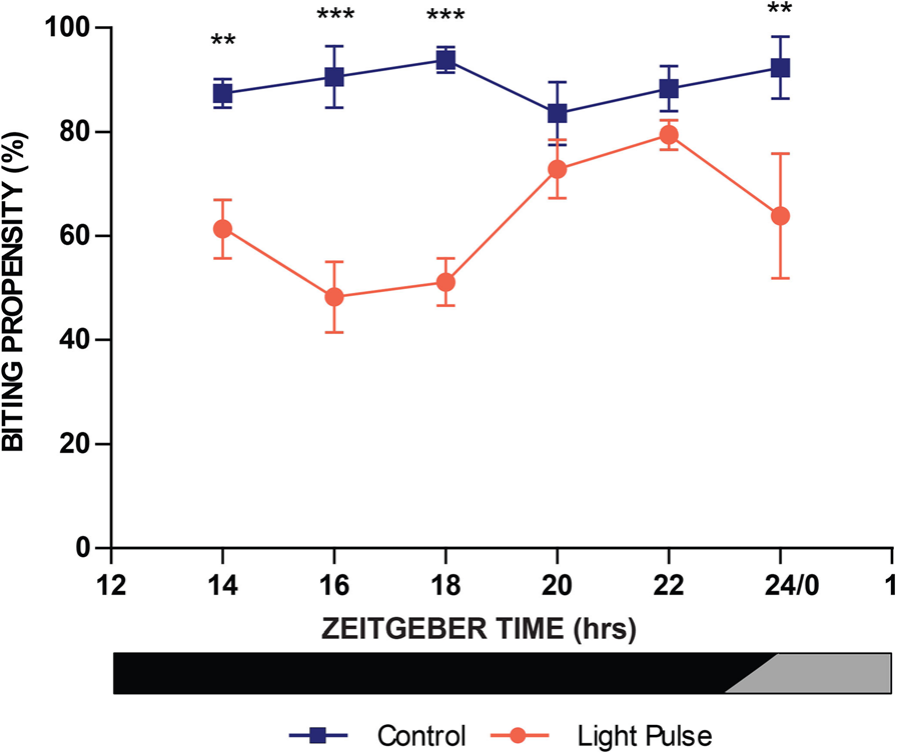
Experiment 4: Sustained (chronic) inhibition of biting behaviour by multiple light pulses delivered during the diel night. Inhibition of blood-feeding in mosquitos that were blood-fed one h 50 min after receiving a light pulse *versus* controls. Light pulses were administered every 2 h throughout the dark phase starting at ZT12. Excluding mosquitoes offered a blood meal at ZT14, experimental mosquitoes were subject to multiple light pulses. Blood meals were offered for 6 min during the biological night at ZT14, 16, 18, 20, 22, and CT24/0 under dark conditions. Two-way ANOVA (effect of treatment, *F*_(1,47)_ = 51.9, *P* < 0.001; effect of ZT, *F*_(5,47)_ = 1.6, *P* = 0.194; interaction, *F*_(5,47)_ = 3.4, *P* = 0.013) followed by *post*-*hoc* tests (*P < 0.05, **P < 0.01, and ***P < 0.001). Values are mean ± SEM

To investigate the sustained biting inhibition effect observed earlier (Fig. 2), and assess its effectiveness as a method to inhibit biting throughout the night, we measured biting propensity every 2 h following precisely-timed light pulses presented every 2 h (Fig. 5). This protocol meant that mosquitoes assessed progressively later into the night would have received an accumulation of light pulses; i.e. mosquitoes assessed at ZT14 received one light pulse, mosquitoes assessed at ZT16 received two pulses, and mosquitoes assessed at ZT18, three pulses, and so on, such that the last population of mosquitoes to be tested for biting at CT24/0 received a total of six pulses. Light-treated mosquitoes displayed a significant decrease in biting propensity compared to controls. Significant differences between treatment and control were detected at ZT14, ZT16, ZT18 and CT24/0, but not at ZT20 or ZT22. While the suppressive effect of light upon biting can be observed at all phases of the night tested (i.e. the mean values for biting propensity are consistently lower than non-pulsed controls), clearly there is a distinct drop-off in efficacy of this sustained effect for 2 h during the late night (ZT20 and ZT22), followed by a return to robust suppression at the start of subjective dawn (CT24/0).

### Experiment 5: Locomotion/flight activity is modified by photic stimuli in an immediate and time-specific manner

Locomotion/flight activity of *An. gambiae* mosquitoes were recorded from individual mosquitoes housed in glass tubes. Data were analysed to determine whether a 30 min light pulse would perturb flight behaviour in mosquitoes acutely (an immediate response) and/or chronically (a sustained/prolonged response). A single light pulse was automatically delivered to the mosquitoes at specific times over the course of the night/dark phase of the LD cycle or during the subjective daytime. In the latter case, at the end of the normal dark phase of the LD cycle, mosquitoes were transitioned into constant darkness, during which a pulse was delivered. At least 3 days of flight activity were recorded including the pulse day and the 2 days prior, and paired comparisons made between these days for individual mosquitoes.

**Fig. 6:**
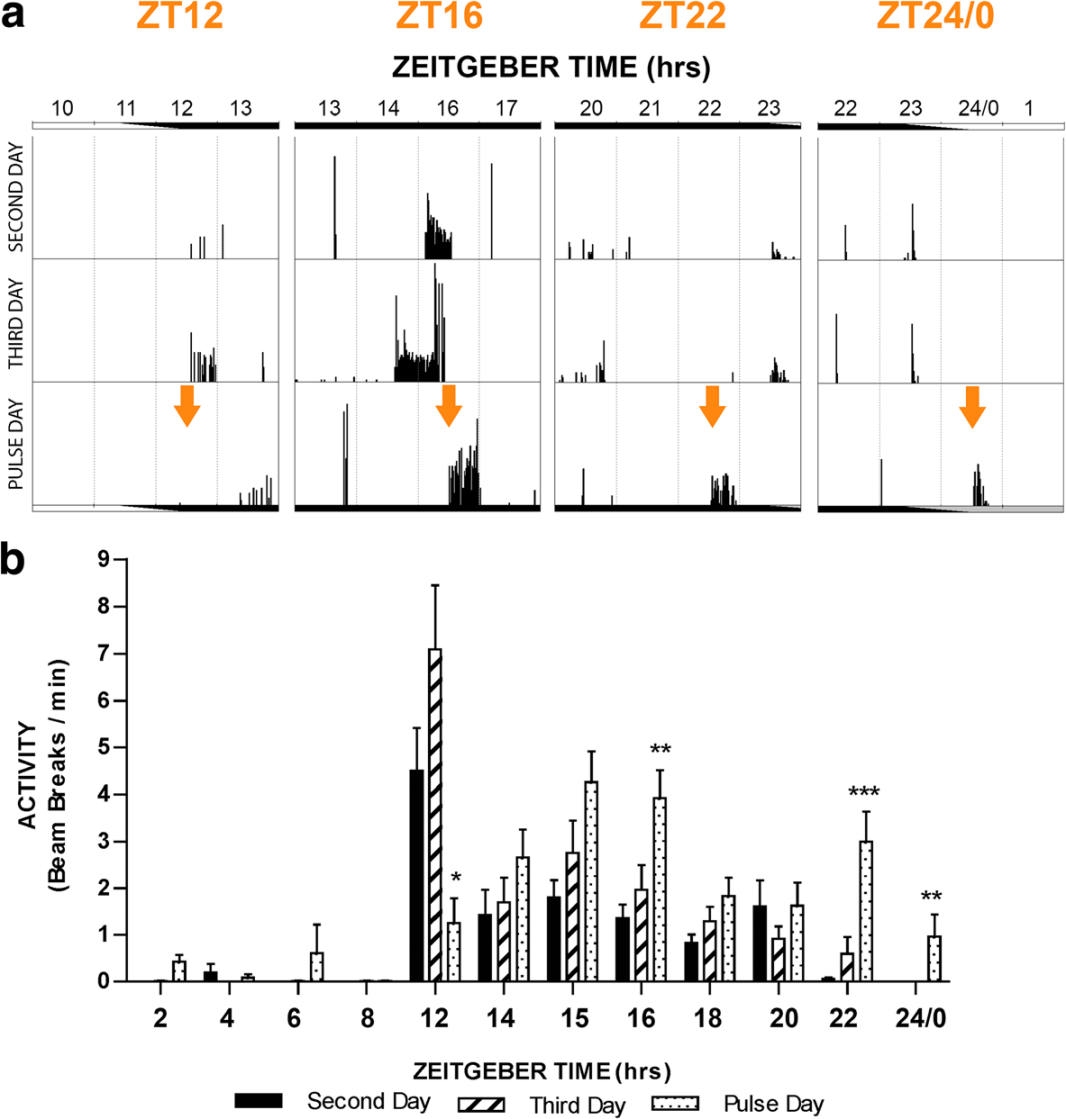
Experiment 5: Light exposure during the night modulates locomotor/flight activity behaviour in a time-specific manner. **a** Representative locomotor/flight activity plots during a 4 h time window of four individual mosquitoes. Mosquitoes were entrained to 12 h/12 h LD conditions (with one h dawn and dusk transitions). Activity during control days (days 2 and 3) under this normal photoperiod are shown (*upper panels*), and on day 4 of the experiment, mosquitoes were exposed to a 30 min, 300 lux white light pulse administered at a precise circadian time (*lower panels*). Control Day 1 is not shown, which was the first 24 h after introduction to the LAM unit. Running from left to right are four individual mosquitoes exposed to light at ZT12, ZT16, ZT22 or CT24/0. The 4 h time window shown for each mosquito is centred on this pulse time. **b** Mean flight activity during a 30 min light pulse delivered at precise Zeitgeber time (ZT)/circadian time (CT) (dotted bars) compared to activity during the same time period on the two prior, non-pulsed days (*black and striped bars*). One-way repeated measures ANOVAs (ZT12, effect of day, *F*_(2,53)_ = 17.9, ***P < 0.001; ZT16, *F*_(2,164)_ = 9.3, ***P < 0.001; ZT22, *F*_(2,65)_ = 14.4, ***P < 0.001; and ZT24, *F*_(2,29)_ = 4.2,*P <0.05) followed by *post*-*hoc* tests (*P < 0.05, **P < 0.01, ***P < 0.001). Values shown are mean ± S.E.M. of individual mosquito activity (*n* = 16–38 mosquitoes per time point). Activity is scored as the average number of LAM beam crossings per minute during the 30 min pulse duration determined for each individual mosquito

Significant changes in flight activity behaviour were observed at four times of the night, specifically at ZT12, ZT16, ZT22 and ZT24/0 (Fig. 6). During the 30 min light pulse administered at the onset of the night at ZT12, mosquitoes displayed a significant decrease in activity compared to both the previous day and the nudiustertian day (i.e. 2 days before the day of treatment). Conversely, when the light was presented later in the night at ZT16, ZT22, or ZT24/0, locomotor activity during the 30 min pulse was elevated compared to both control days (Fig. 6). No significant differences in activity were observed during the 30 min pulse when administered during the phases that fell during the subjective day of the circadian cycle.

**Fig. 7:**
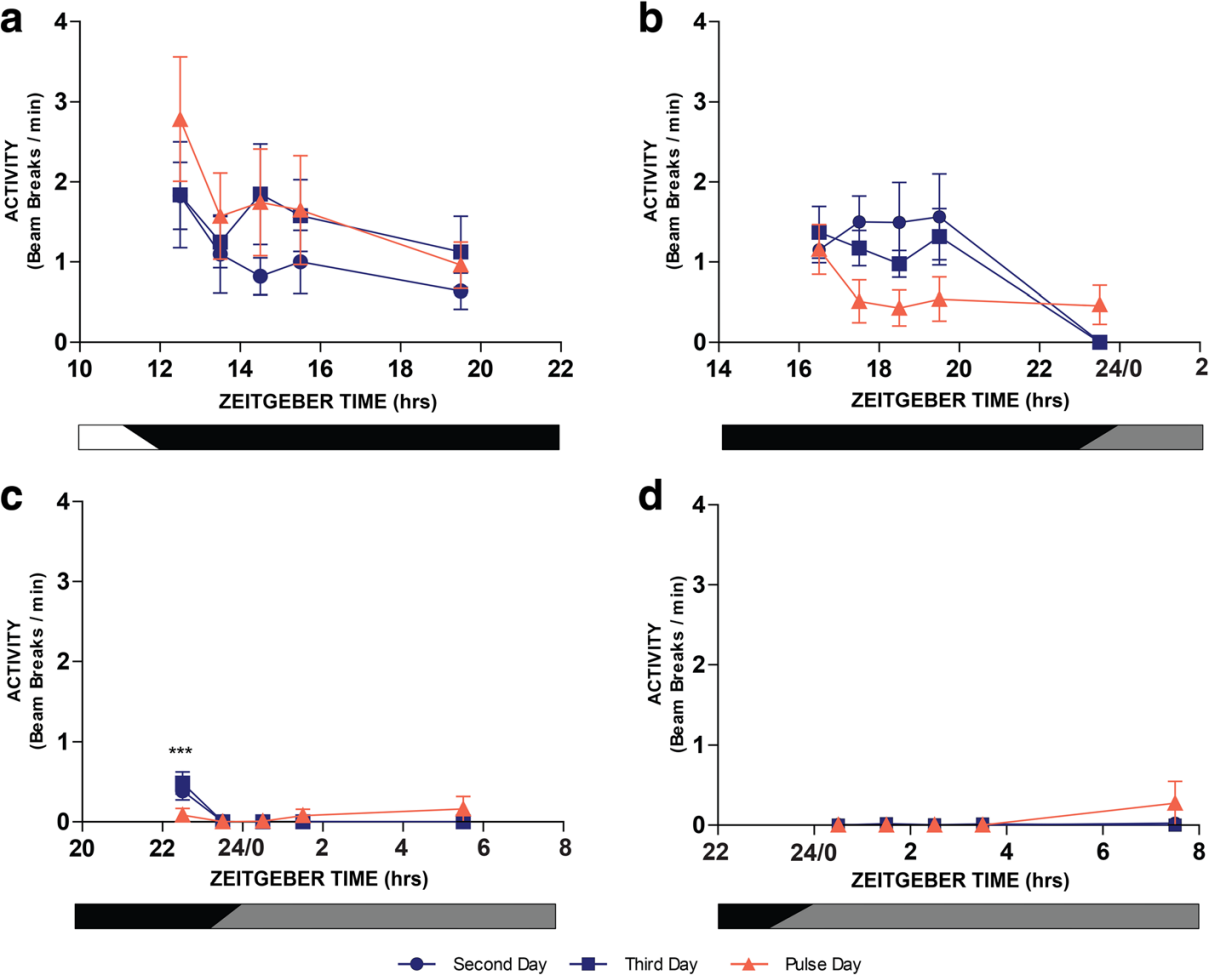
Sustained effects of a 30 min light pulse on *An. gambiae* mosquito flight activity. Mean flight activity measured during a 60 min period at various intervals over 8 h (1, 2, 3, 4 and 8 h), following exposure to a 30 min light pulse. Analysis was conducted at times of the circadian cycle that correspond with changes in activity that were detected *during* the 30 min exposure to light, i.e. at ZT12 (**a**), ZT16 (**b**), ZT22 (**c**) and CT24/0 (**d**) (see also Fig. 6 for mean flight activity measured during the light pulse administration). Mean flight activity is compared on the day of treatment (triangle symbols) to activity during the same period on the two prior, non-pulsed days (circle and square symbols). A pulse administered at ZT22 significantly inhibited activity during 1 h following the pulse (i.e. at ZT22.5–23.5; Table 1). No other significant differences between treatment and control days were observed during the 8 h following the time-specific light pulses Table 1). One-way RM-ANOVAs followed by *post*-*hoc* tests (***P < 0.001). Values shown are mean ± S.E.M. of individual mosquito activity. Activity is scored as the average number of LAM beam crossings per minute during the 60 min duration determined for each individual mosquito at the specified time intervals

We next explored whether the light treatment would lead to any sustained effects on flight activity. We, therefore, measured activity over a 1 h duration starting at different time intervals following the end of the light pulse. Despite the acute effect seen from light pulses administered at ZT12, ZT16, and ZT24/0, there were no significant sustained perturbations in locomotion/flight activity observed at 1, 2, 3, 4, or 8 h post-pulse following these acute effects (Table 1: Fig. 7a, b and d); however, statistical analysis revealed a sustained decrease in activity for 1 h post-pulse (i.e. ZT22.5-23.5) when the pulse was administered during the late night at ZT22 (Table 1; Fig. 7c).

**Table 1:** Analysis of sustained/chronic changes to flight activity examined over 8 h following light pulse treatment (repeated measures ANOVA analysis)

Treatment time	Repeated measures two-way ANOVA		
	Effect of day	Effect of ZT	Interaction
ZT12	*F*_(2,269)_ = 2.2, *P* = 0.110	*F*_(4,269)_ = 2.3, *P* = 0.056	*F*_(8,269)_ = 0.281, *P* = 0.972
ZT 16	*F*_(2,824)_ = 1.8, *P* = 0.169	*F*_(4,824)_ = 2.7, *P* = 0.032*	*F*_(8,824)_ = 1.6, *P* = 0.112
ZT 22	*F*_(2,344)_ = 0.3, *P* = 0.774	*F*_(4,344)_ = 11.7, *P* < 0.001***	*F*_(8,344)_ = 2.9, *P* = 0.004**
ZT24/0	*F*_(2,149)_ = 0.7, *P* = 0.503	*F*_(4,149)_ = 1.1, *P* = 0.374	*F*_(8,149)_ = 1.0, *P* = 0.464

*P < 0.05, **P < 0.01, ***P < 0.001

## Discussion

In this study, we have explored how light can modify *An. gambiae* blood-feeding behaviour. We used timed white light treatments under controlled laboratory conditions presented singularly or as multiple pulses at various intervals throughout the night, to explore the efficacy of light to reliably suppress adult female mosquito biting and modify flight activity. The current work builds upon our earlier studies of time-of-day-specific blood-feeding activity in *An. gambiae* mosquitoes, and how temporal changes in behaviour correlate with physiological and molecular changes in olfactory modalities [20]. Using a human arm blood-feeding assay, the Pimperana strain exhibits a 24 h rhythm in biting, peaking during the early night. This rhythm is preserved under constant conditions of darkness, thereby revealing that the rhythm is also driven, at least in part, by the endogenous circadian clock of the mosquito [19, 20]. The experiments in the current study also complement the work of Das & Dimopoulos [19], that explored biting suppression in an outbred strain during the late night using short pulses of bright light (2 min, 800–1000 lux) and with an artificial membrane feeder in the absence of a human [19].

The current study was designed first to explore the potential modulation of blood-feeding behaviour by light specifically during the early night and late daytime, times of the 24 h day that are proving to be critical in residual biting activity even when ITNs have been introduced to a malaria endemic area. Secondly, on establishing an efficient and effective level of suppression during the early night, we wished to determine if the immediate suppressive effect could be reproduced at all times of the night. Thirdly, on discovery of a long duration (2–4 h) chronic suppression of biting following a single pulse of light, and to develop an efficient method to suppress biting activity throughout the night, mosquitoes were exposed to multiple short, discrete pulses of light. To complement the biting studies, and since flight activity is a component of host-seeking behaviour [26], we tested for modulatory effects of a light pulse treatment presented at different times of the 24 h day upon a quantitative measure of general mosquito flight activity.

Anopheline mosquitoes are predominately nocturnal, concentrating their feeding and flight activity (swarming, migration and host-seeking) to the night [12, 13, 16, 20, 27]. We, therefore, hypothesised that a light pulse would inhibit or reduce the propensity to bite a human host when administered during the dark phase of the LD cycle. In Experiment 1 of our investigation, *An. gambiae* mosquitoes demonstrated an acute (tested at ZT12:10) and sustained (tested at ZT14) inhibition of biting activity after a receiving a single 10 min white light pulse presented at ZT12. Remarkably, the level of biting suppression assayed on the human arm immediately after cessation of the light pulse was at an average of 42% of biting control levels. This inhibition remained high at 32% for 2 h after delivery of the light pulse; and somewhat surprisingly, the photic effect upon biting behaviour in some trials was sustained for up to 4 h after the pulse.

The results of our second experiment in which we examined the potential for light exposure during the late daytime to suppress biting activity were as predicted from our previous work. In Rund et al. [15] using the same strain of mosquitoes, we had observed that the biting propensity of mosquitoes studied under diel conditions to be close to 0% during the day phase of the LD cycle [20]. However, under constant dark (circadian) conditions, while a distinct 24-h rhythm persisted for several days, the level of biting during the ‘subjective’ daytime was elevated up to 70% of maximal night-time biting levels. We, therefore, hypothesised that the presence of light during the daytime has a direct suppressive effect on the biting behaviour of the mosquito. Furthermore, in a limited experiment, the effect of a 15 min pretreatment of darkness on biting activity was examined and revealed an elevation when tested at each of three different daytime phases of the diel cycle (ZT0, ZT4 and ZT8) [20]. To examine this phenomenon in a more controlled and complete fashion, we tested the effect of a 15 min treatment of darkness presented during the late daytime (ZT9.25) or during dusk (ZT11.25) before undertaking the standard blood-feeding assay. The results of this procedure revealed a distinct elevation in biting activity as compared to time-matched control mosquitoes that had simply been exposed to light within the normal LD cycle. The effect was potent, as the dark-treatment during the late daytime (2 h prior to dusk or during actual dusk) resulted in a 58% elevation in biting propensity compared with control mosquitoes. Moreover, this effect was observed and equally effective at both times of the late daytime that were tested. This is an important finding since it suggests that while *An. gambiae* mosquitoes concentrate their blood-feeding during the night, and that this behaviour is driven by their endogenous circadian clock [19, 20], there is a residual negative masking effect of light that occurs during the daytime.

In the third experiment, we tested the immediate response of the mosquitoes to light while simultaneously offering a human blood meal. These tests were performed at one of six different times of the night to evaluate whether differential responses would occur that were time-of-day dependent. By testing at 2 h intervals, this immediate inhibition effect was found to occur during the entire night, i.e. at times tested during the early to late night (i.e. ZT12-ZT22), and resulting in a dramatic suppression of biting activity of 42–66% as compared to the time-matched controls. Interestingly, a clear incremental decline of suppression was observed as the night progressed. The differential responses that were time-of-day specific suggest an underlying circadian property of the mosquito physiology that results in the altered treatment efficacy.

Based on the observations of these experiments, we expanded this approach to photic manipulation of the mosquitoes to develop a method to reduce biting propensity throughout the night by exposing mosquitoes to a series of light pulses presented every 2 h. We hypothesised that this protocol would result in a sustained inhibition of blood-feeding behaviour throughout the duration of the night. In this fourth experiment, the protocol elicited a sustained suppression of biting activity that was observed during the early to middle of the night, as well as at the very end of the night/dawn (ZT14, 16, 18 and CT0/24). While statistically significant differences were not observed for ZT20 and ZT22, the means for the pulsed groups were lower than the time matched control groups. This was somewhat surprising as we had hypothesised that the sustained response would be equal at all times of the night. However, these data suggest in a manner similar to the immediate responses tested in experiment 3, that the sustained effects of light are influenced by an underlying circadian property of the system. Additionally, the change in biting propensity may reflect an increased homeostatic drive to blood feed as the night progresses that then competes with the light-suppressive mechanism. However, despite the reduced efficacy of light delivered during the middle of the night, the sustained response using the multiple pulse approach provided suppression of biting during the early to middle night and late night/dawn phases of the night. As these times of night are critical phases of the diel cycle when humans are most susceptible to biting events as they are unprotected when not sleeping under a bed net, the results of the experiment suggest that indeed this multi-pulse method might be effective in the control of mosquito biting events in the field.

These striking results on photic manipulation of *An. gambiae* blood-feeding behaviour generates several questions regarding the mode of action by which photic cues impact mosquito physiology and behaviour. It is likely that the mechanism within the brain circuitry for the acute behavioural response (suppression of biting) to light is mediated by a neuronal response (potentiation and integration) and/or phosphorylation of protein(s) due to its rapid response; whereas the sustained effect of photic stimulation is likely to result from the turnover of protein and/or *de novo* change in gene expression and subsequent change in protein synthesis [28, 29]. Such changes in gene expression have been observed in *Drosophila* head and *An. gambiae* head and whole body following photic stimulation [19, 30, 31]. However, it is unlikely that the behavioural effects observed in the current study are operated through changes in the circadian clock [14, 32]; yet in theory, a phase shift of the clock could change the temporal profile of the 24 h biting preference rhythm [20]. The clock in other organisms has been shown to be reset by light by 2–3 h but not at 1 h after the start of a precisely-timed photic stimulus [33, 34]. Therefore, a shift of the circadian clock would not explain the acute suppression of biting that is observed immediately after the start of the light pulse (i.e. within 15 mins at ZT12.25), or during the actual exposure to the light, as observed at various times during the night. A shift of the clock might, however, contribute to the changes observed after two or more hours after photic treatment (e.g. at ZT14 when treated with light for 10 min at ZT12). As light presented during the early night (ZT12–14) would be predicted to produce a phase delay of the clock and light during the middle to late night (ZT15–24/0) would result in a phase advance [19, 35], these adjustments may well shift the position of the biting rhythm [19, 20]. These shifts of the clock, based on a circadian light phase response curve (PRC) for *An. gambiae* [35], might explain some but not all of the results of Experiment 4, where mosquitoes were exposed to multiple pulses of light at night. We would predict to generate relatively small shifts of the clock, and maximally a 2 h average shift when the light is presented at ZT14 (delay) or ZT15 (advance). However, since the average biting propensity of the control groups of mosquitoes in Experiment 4 was ≥ 80% when examined at different times across the night, and the biting activity of light-pulsed mosquitoes tested at ZT16 and ZT18 was ≤ 50%, factors other than a shift of the endogenous biting rhythm would have to account for this suppression of biting. The exception would be a discrete light pulse delivered at ZT22 and tested 2 h later at CT24/0 since a phase advance might shift the biting rhythm into the subjective day, and thus to a phase of the circadian cycle where we would expect reduced biting activity [19, 20].

While not explicitly tested in the current investigation, we hypothesise a dose dependency of photons per unit time, such that a higher light intensity would require a lower exposure duration to reach the desired level of suppression of feeding [19]. For example, 1 min at 5000 lux may provide the equivalent inhibition of feeding as 10 min at 300 lux and 30 min at 30 lux. Therefore, it is plausible that an increased dose of light could unmask some of the subtle effects of light upon suppression, such as at ZT20–22 in the multi-pulse experiment, as well as simply increase the treatment efficacy at all phases of the night and late daytime. A similar prediction could be made for a dependency upon the specific wavelength(s) of light upon the efficacy of the pulse treatment [36, 37]. We predict that there will be a specific wavelength(s) of light that will have maximal efficacy in the immediate and sustained suppression of biting behaviour and related effects of suppression or elevation of flight activity. Selecting a monochromatic light source in this maximal range would allow for modulation of behaviour at lower intensities of light. Additionally, selecting a monochromatic light source that is outside of the blue colour of the spectrum, even if sub-optimal, might allow for manipulation of mosquito behaviour without disruption of human behaviour/physiology (e.g. human arousal, circadian phase shifting) [38, 39]. In the current study we purposefully used broad-spectrum white light, but having established a baseline level of suppression, testing different colours of light might allow for enhanced utility of light as a vector control method. Ultimately, testing this variable as well as different light intensities and durations of exposure is beyond the scope of the current study.

In an effort to understand the broader impact of light on *An. gambiae* behaviour, we examined mosquito flight activity during and several hours following treatment with white light at different phases of the circadian cycle including both the night and subjective daytime (tested while in constant darkness). Adult female mosquitoes exposed to the photic treatment demonstrated an acute flight inhibition during a 30-min light pulse when presented at the start of night at ZT12 (a 79% suppression of activity compared to the same Zeitgeber time of control days), and conversely an acute elevation of flight activity during an equivalent light pulse presented at ZT16, ZT22 or CT24/0 (with a dramatic ≥ 1000% increase of activity when the mosquito was treated at the end of night or dawn). There was also a sustained effect on flight activity during the 1 h immediately following cessation of the light when the administration of the light pulse was during the late night at ZT22; however, the activity was reduced (by 80%), not increased as observed during the time of pulse administration. In the time-specific light pulse treatment groups examined (namely ZT12, ZT16, ZT22 and CT24/0), this was the only significant change in flight activity detected over the course of the 10 h following the photic treatment.

We propose that the sudden inhibition of flight activity seen at ZT12, and elevation of activity at ZT22 and ZT24, represent ‘masked’ responses [40] and comparable to the immediate suppression of biting activity during exposure to the light pulse, as briefly discussed above. In this mechanism upon the area becoming illuminated the mosquito behaviour is acutely altered in a negative or positive manner, and that a shift in the circadian timing system is not directly responsible for the immediate behavioural response. Negative, positive and paradoxical masked responses to light have been documented in a similar fashion in laboratory rodents and *Drosophila* [40–42]. Furthermore, complementary to the data presented here, modifications to swarming behaviour of anophelines in response to changes in light exposure have been reported in the field and laboratory [36, 43]. For example, when located inside a dark outdoor barn, male *An. maculipennis atroparvus* demonstrated typical swarming behaviour; however, when the light intensity of the area increased by ~1.0 log lux, swarming abruptly stopped [43]. Interestingly, when the light was prevented from entering the area swarming recommenced. This suggests that the acute inhibition of activity by light was, like the data we present here, a masked response to the stimulus and not an endogenous response mediated by the circadian clock. Masked responses may provide increased fitness benefits to the organism by allowing a rapid reaction to environmental change. For example, upon the sudden illumination of an occupied area, survivorship is increased (e.g. by avoiding potential predation or desiccation) [44]. As clock-mediated adaptation takes considerably longer to manifest (> 1 h), it would be evolutionary detrimental for a clock mechanism to orchestrate such responses.

The result at ZT12 of photic suppression of flight activity is perhaps not surprising. The intense and short-lived elevation in general activity that normally corresponds with dusk/onset of night is likely related to behaviours such as the movement from resting sites, swarming, migration and sugar feeding [12, 16, 27]. An interaction of environmental variables such as light and endogenous rhythms can be seen in a modification to the profile of flight activity at dusk. Also studied using the LAM unit assay, both *An. gambiae* and *An. coluzzii* exhibit a different minute-to-minute pattern of activity depending on whether mosquitoes are exposed to a ‘natural’ dusk transition or maintained under conditions of constant darkness [16]. As has been demonstrated in various mosquito species including anophelines, the light intensity is an important factor in the timing of swarming events [12, 36], such that intensities above certain thresholds can result in cessation of swarming or inhibition of its onset.

While we believe that the current study is the first to quantify changes in flight activity of *An. gambiae* mosquitoes over the 24-h circadian cycle, Jones et al. [35] qualitatively described a “lights on reaction” in *An. gambiae* flight activity when exposed to light under DD conditions and using an acoustic assay [35]. Consistent with the current investigation, a decrease in activity was observed at ZT12, marked increases when the light was presented later in the night, and no responses observed during the subjective daytime. The consistency of observations between studies despite using different experimental approaches suggests that the acute responses to photic stimuli represent a highly reproducible behavioural response that is conserved across *An. gambiae* strains.

From the experimental design of the study used here, it is unclear if the observed increases in flight activity observed during the night and dawn were photophilic, photophobic or reflect an escape response. The modulation of locomotion/flight activity in our LAM assay could represent positive or negative phototaxis, such as an attraction to the exit of a shelter, or a shelter-seeking ‘escape’ response [45]. Alternatively, it could be a neutral phototaxic response, i.e. no specific orientation, and simply represents an increase or decrease in generalised flight activity, e.g. the photic suppression of activity that we observe with a treatment at ZT12 could represent a suppression of swarming-related ‘excited’ activity. However, without further experimentation, using an arena apparatus, for example, it is unclear what the suppressed (at ZT12) or elevated flight activity (at ZT16, ZT22 and ZT24/0) represents in the natural behavioural repertoire of the mosquito.

The mechanism by which the photic cues are sensed by the mosquito, that in turn influence brain-behaviour pathways involved in the modulation of biting and flight activity, is likely to include the photoreception cascade of the compound eye [46, 47]. The use of broad spectrum white light does not favour or preclude one specific opsin photopigment or class of ommatidium involved in detecting blue, green or red wavelengths of light [14, 47, 48]. The immediate response to photic stimuli are indeed likely to be conveyed by the compound eyes and *via* their neuronal connections to the brain, but it is plausible that the sustained effects of the light treatment involve non-retinal photoreception. This might include the flavoprotein cryptochrome photopigment system (specifically *CRY1* in *An. gambiae*), found expressed throughout the insect body [14, 49], and that is important in the photic-resetting mechanism of the insect circadian clock [49–53]. Obviously identifying the specific opsins, photoreceptors, and downstream neuronal and endocrine pathways associated with the behavioural responses reported herein generates interesting questions for future investigation.

We note this is a laboratory-based study, using one inbred mosquito line, with mosquitoes kept in small containers, and shielded from any low-level nocturnal light and from continuous exposure to host odours. To complement any field-trials, future laboratory work might include a determination of the optimal wavelength of light to produce a given behavioural response, generate a photic dose response curve, optimize the spacing and timing of light pulses, and explore further the effect of low-level nocturnal light, e.g. moonlight, on behaviour [54–56]. The suppressive nature of light on mosquito biting on successive and subsequent nights of treatment, and in the constant presence of host cues might also be aspects worthy of evaluation.

## Conclusions

In conclusion, we find that light is a potent modulator of mosquito biting behaviour and flight activity. These data provide evidence for both immediate (acute) and sustained (chronic) suppression of biting behaviour. Photic suppression of biting shows greatest efficacy during the early and mid-part of the night and the late daytime. Flight activity, a component of host-seeking behaviour, is decreased by light at night onset and increased during the middle of the night and at night offset: this could represent photophilic, photophobic or photo-neutral behaviour. Furthermore, time-of-day specific differential responses in biting behaviour and flight activity suggest an underlying circadian property of these biological systems. Finally, multiple pulses of light with long 2 h intervals could be an efficient method to suppress biting activity, especially indoors: This could augment current barrier and insecticidal strategies used to control mosquito-human interaction.

At present, insecticide-treated bed nets and indoor residual spraying of insecticides are heavily relied upon to prevent the transmission of malaria; however, as mosquitoes are becoming increasingly resistant to insecticidal treatments, and *Plasmodium* spp. become resistant to drug treatments [3, 57], there is a necessity for the ongoing development of novel and innovative control strategies. The current data illustrates the development of a method for the manipulation of mosquito biting and flight/locomotor activity behaviours using exposure to white light presented at timed intervals or during the late daytime, dusk, dawn, and during the night. This treatment may provide a useful method to augment current insect control methods, as well as acting alone, to reduce host-seeking (which involves flight) and biting events.

## Abbreviations

CT: Circadian time
DD: Dark:dark
IRS: Indoor residual spraying
ITN: Insecticide-treated net
LAM: Locomotion activity monitor 25
LD: Light:dark
PRC: Phase response curve
ZT: Zeitgeber time
